# Genetic analysis of the equine orthologues for human CYP2D6: unraveling the complexity of the CYP2D family in horses

**DOI:** 10.3389/fvets.2023.1188633

**Published:** 2023-10-19

**Authors:** Giada Scantamburlo, Charity Nofziger, Markus Paulmichl, Simone Vanoni

**Affiliations:** ^1^Pharmgenetix GmbH, Niederalm-Anif, Austria; ^2^Privatklinik Maria Hilf GmbH, Klagenfurt, Austria

**Keywords:** horse pharmacogenetics, CYP2D6 equine orthologues, CYP2D50, CYP2D82, drug metabolism

## Abstract

**Introduction:**

Because of their importance as companion animals or as racehorses, horses can be treated with various drugs. Although it is known that drug withdrawal times can vary for each horse, pharmacogenetics for these animals has not been adequately studied and requires further development. Since *CYP2D6* is responsible for the metabolism of 25–30% of drugs in humans, including some used to treat horses, a study of the *CYP2D* family in horses was conducted to define its genetic structure as well as its expression pattern in the liver.

**Methods:**

Genomic DNA extracted from venous blood and mRNA from fresh liver tissue were amplified and sequenced to analyze the genomic structure, genotype, and expression of the various enzymes that are part of the equine orthologous family for *CYP2D6*.

**Results:**

Amplification and sequencing of the gDNA of *CYP2D50*, the major *CYP2D6* orthologue identified in previous studies, revealed a novel putative genomic structure for this gene compared with that reported from the EquCab3.0 assembly, including the formation of a hybrid structure similar to what happens in human *CYP2D6*. At the mRNA level, transcripts from six different members of the equine *CYP2D* family were detected in horse liver. In addition, genotyping of *CYP2D50* and *CYP2D82* revealed the presence of several polymorphisms, six of which result in novel, nonsynonymous amino acid changes for each of the two genes.

**Discussion:**

This study aimed to elucidate the pharmacogenetic analysis of the *CYP2D* family in horses and resulted in the identification of a novel gene structure for *CYP2D50*, the expression of six different members of the *CYP2D* family in horse liver, and several novel polymorphisms for *CYP2D50* and *CYP2D82*.

## Introduction

Pharmacogenetics has become an increasingly important topic in recent years, driving medicine toward more personalized therapy. Genetic variations can greatly affect an enzyme’s ability to metabolize drugs, and by identifying these functional enzymatic variations, a more efficient therapy can be prescribed (1). The acquisition of pharmacogenetic knowledge is not limited to human genetics but is also echoed in veterinary medicine. Horses are among the companion and sport animals that can be widely treated with drugs such as NSAIDs (2–4), corticosteroids (5, 6), local anesthetics (7, 8), mucolytics (9), sedatives (10), antibiotics (11–13), diuretics (14–16) and PPIs (17, 18). In addition to certain medications that are banned in racehorses, others may be administered as needed, but are subject to control through drug testing. The Federation Equestre Internationale (FEI) provides a List of Detection Times (19) that indicates the approximate length of time that a drug or its metabolite remains in a horse’s system and is therefore detectable. This document also notes that the withdrawal time for a drug should not be decided simply on the basis of the stated detection time, but that individual differences between horses such as size, fitness level, metabolism, and concurrent administration of other drugs should be considered. Genetic variations that impair the functionality of enzymes involved in the metabolism of these controlled drugs can result in positive doping findings even when standard drug withdrawal is followed, leading to regulatory problems for racehorses (20, 21). Considering that the annual economic value of the combined sport horse and breeding industry in Europe is 52.1 billion euros per year (22), pharmacogenetics may become an important turning point in the pharmaceutical treatment of horses.

The cytochrome P450 (CYP450) superfamily of monooxygenase enzymes is well known in humans and is responsible for various reactions involved in life processes, mainly drug metabolism and detoxification of xenobiotics, but also for the synthesis of endogenous molecules such as steroids or vitamin B3 (23). Cytochrome 2D6 (*CYP2D6*), which is responsible for the metabolism of 25–30% of currently available drugs, including antiarrhythmics, antidepressants, chemotherapeutic agents, and opioids, is one of the most important members of the family in humans, although it accounts for only 2% of the total CYP content in the liver (24, 25). One of the main features of *CYP2D6* is that it is highly polymorphic, leading to high interindividual variability in its activity. Accordingly, these differences are reflected in four different phenotypes, termed Ultrarapid (UM), Extensive (EM), Intermediate (IM), or Poor (PM) metabolizers (26). The clinical relevance of these specific phenotypes is high, especially for UM or PM, which can lead to adverse drug reactions or impaired treatment efficacy, depending on the metabolic pathway of the drug (27). These four phenotypes have also been observed in horses (28, 29). Several drugs that are metabolized by *CYP2D6* in humans, such as dextromethorphan, lidocaine, codeine, or tramadol, can also be administered to horses for the treatment of crib-biting (30), as cough suppressants (31), or for general analgesia (32, 33). In addition, the risk of accidental exposure to drugs or substances that test positive in racehorses is high (34, 35), even considering that dextromethorphan, its metabolite dextrorphan, and other common human therapeutics that use the same hepatic pathway, such as tramadol and venlafaxine, are frequently present and stable in the environment, with a peak during the cold season (36). However, to date, knowledge of the equine orthologues for *CYP2D6* is limited, as is the presence of possible polymorphisms leading to impaired drug metabolism, although some studies indicate metabolic variability in equine CYP2Ds (29, 37). In contrast to humans (where only one member of the CYP2D family is active), but similar to other species such as rodents or rabbits, a total of six CYP2Ds have been identified in horses (38). To date, only two of these genes, *CYP2D50* and *CYP2D82*, have been shown to be responsible for the metabolism of dextromethorphan (28) and codeine (29, 39), respectively. Moreover, similar to humans, CYP2Ds in horses and their polymorphisms can have strong clinical effects on drug metabolism, resulting in adverse effects or lack of efficacy (29). However, little is known about the expression and activity of all other CYP2Ds in horses (38). Given the importance that pharmacogenetic may have in horses, the aim of this study is to analyze the genetic structure and liver expression patterns of *CYP2D6* equine orthologues and to define the presence of polymorphisms in these genes that may lead to functional effects.

## Materials and methods

### Sample collection

Venous whole blood samples were collected in EDTA K Monovettes® (Sarstedt; Wr. Neudorf, Austria) by venipuncture from 71 horses (4 Noriker, 1 Quarter, 1 Icelandic, and 51 Warmblood) at the Tillysburg Pferdeklinik in Sankt Florian (Austria). Fresh liver tissue was also collected immediately after euthanasia from 4 of these horses (1 Noriker, 1 Quarter, and 2 Warmblood). 0,5 cm cubes were cut from the fresh tissue and immediately submerged in RNA Later stabilization reagent (Qiagen, Hilden, Germany). Transport was performed at 4°C.

### Genomic DNA (gDNA) isolation from whole blood samples

gDNA was isolated from ~350 mL blood using the EZ1 DNA Blood 350 mL Kit (Qiagen, Hilden, Germany) on the EZ1 Advanced XL platform (Qiagen, Hilden, Germany) according to the manufacturer’s instructions with an elution volume of 50 μL. gDNA was quantified with the QIAxpert (Qiagen, Hilden, Germany) spectrophotometer. Only samples with an A260/A280 between 1.700 and 1.900 were used for downstream analyses.

### mRNA isolation from liver tissue and reverse transcription

mRNA was isolated from 60-65 mg of liver tissue stabilized with RNAlater using the QIAamp RNA Blood Mini Kit (Qiagen, Hilden, Germany) according to the manufacturer’s instructions with an elution volume of 30 μL. The tissue was disrupted using a mortar and pestle and then homogenized using a QIAshredder homogenizer. The mRNA was quantified with the QIAxpert (Qiagen, Hilden, Germany) spectrophotometer. Only samples with an A260/A280 between 1.900 and 2.000 were used for downstream analyses. mRNA was reverse transcribed using the QuantiTect Reverse Transcription Kit (Qiagen, Hilden, Germany). 1 μg RNA was incubated with 1X gDNA wipe-out buffer in an 84 μL reaction for 2 min. at 42°C, followed by 2 min. cooling on ice. 41.5 μL of the incubated mixture were then added to 18 μL + RT and -RT reactions. Both these reactions contained 1X Quantiscript RT buffer and 3 μL RT primer mix. 3 μL Quantiscript reverse transcriptase was added to the +RT mix and 3 μL RNAse-free water was added to the -RT mix. The resulting 60 μL reactions were incubated for 30 min. at 42°C, followed by 3 min. at 95°C and cooling on ice.

### PCR and sanger sequencing

PCR reactions (50 μL) were prepared using JumpStart REDAccuTaq LA polymerase (Sigma, St. Louis, Missouri) and consisted of 1X AccuTaq LA Buffer, 0.5 mM dNTPs, 5% DMSO and 0.4 μM forward and reverse primers. The PCR parameters for amplification of transcripts are 96°C for 30 s. followed by 35 PCR cycles with denaturation at 94°C for 15 s., 30 s. annealing and elongation at 68°C for 1 min. 50 s. followed by an additional elongation at 68°C for 30 min. For the amplification of genes, the parameters are 96°C for 30 s. followed by 35 PCR cycles with denaturation at 94°C for 15 s., 30 s. annealing and elongation at 68°C for 4 min. 45 s. followed by an additional elongation at 68°C for 30 min. Primers sequences and specific annealing temperature for the different targets are listed in Supplemental Table S1. The presence of the expected amplicon was verified on a 0.5% agarose gel. The PCR product was purified using the QIAquick PCR purification kit (Qiagen, Hilden, Germany) according to the manufacturer’s instructions. The amplicon was sequenced by Sanger sequencing (Microsynth, Balgach, Switzerland) in the forward and reverse orientations with the following primers for each specific target: CYP2D50–1 transcript (211, 212, 220, 221, 222, 223, 331), CYP2D50–1 gene (220, 221, 222, 223, 228, 265, 266, 267, 254, 331), CYP2D50–2 transcript (220, 221, 223, 228, 253, 331), CYP2D50–2 gene (220, 221, 223, 228, 231, 265, 266, 267, 253, 331), CYP2D82 transcript (395, 396, 397, 398), CYP2D82 gene (395, 396, 397, 398, 507, 508, 509, 510, 511, 512, 513, 514). For sequencing primers sequence, see Supplemental Table S2. For the other CYP2D transcripts, the sequencing was performed using the PCR primers. Sequencing results were analysed using MacVector Software version 18.1.3.

### Real-time PCR

10 μL real-time PCR reactions were performed using SYBR Green in white 384-well plates on the Quanstudio 12 K Flex instrument (Thermo Fisher Scientific, United States). Each reaction consisted of 1X SYBR Green (Thermo Fisher Scientific), 250 nM forward and reverse primers and 1:10 diluted cDNA or -RT reaction. Primer sequences for the different targets are listed in Supplemental Table S1. Cycling parameters were as follows: an initial denaturation at 55°C for 2 min. and 95°C for 2 min., followed by 40 cycles at 95°C for 15 s., 55°C for 15 s. and 72°C for 1 min. At the end of the cycles, a melting curve step was added to detect the formation of non-specific products. Gene expression values were determined using the relative standard curve method and actin as the reference gene. GraphPad Prism Software version 9.1.1 was used to plot the values and for the unpaired t-test with Welch’s correction for statistical analysis.

### Protein function prediction tools

Sorting Intolerant From Tolerant (SIFT) (40, 41) and PROtein Variation Effect ANalyzer (PROVEAN) (42, 43) were used as protein function prediction tools. For both tools, the *wild-type* protein sequence was submitted in FASTA format along with a list of amino acid substitutions as described on the website. In SIFT, the analysis parameters were kept as default, and a single SIFT score from 0 to 1 was determined for each of the submitted substitutions (where 0 is considered the most deleterious and 1 as fully tolerated), with all substitutions with a score below 0,05 considered deleterious. For PROVEAN, the threshold score was left at the default value (−2.5) to ensure consistency in sensitivity and specificity, with all amino acid substitutions falling below a score of −2.5 defined as harmful.

## Results

### Identification of a new genetic structure for CYP2D50 gene

**Figure 1:**
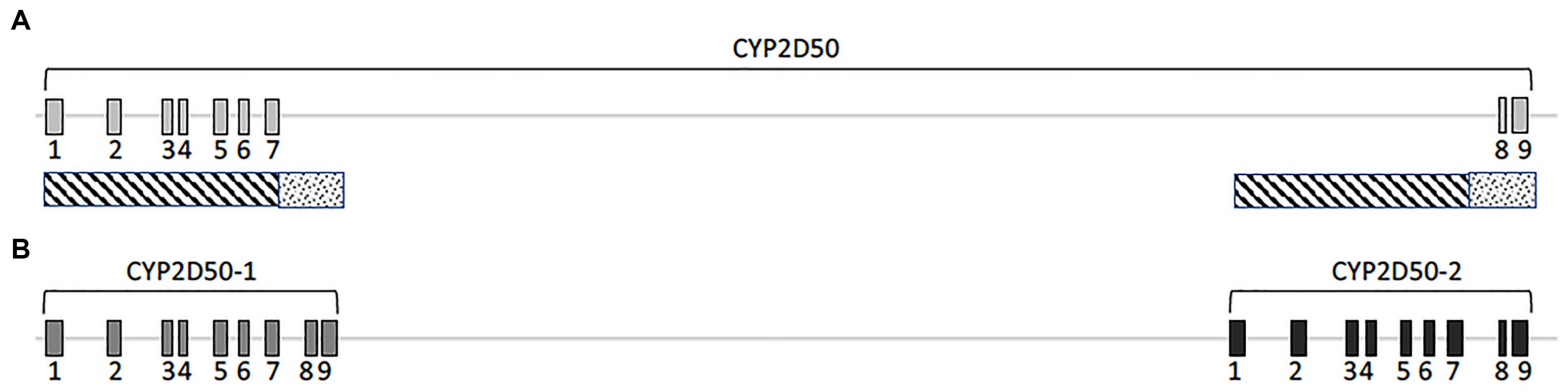
Cartoon of the CYP2D50. **(A)** Original genetic structure as proposed in the EQUCab3.0 assembly and **(B)**. Two genes structure as proposed in this study. The line represents the genomic sequence, while the numbered boxes represent the exons. The dashed and dotted boxes under **(A)** indicate the two regions that have a high degree of identity.

**Figure 2:**
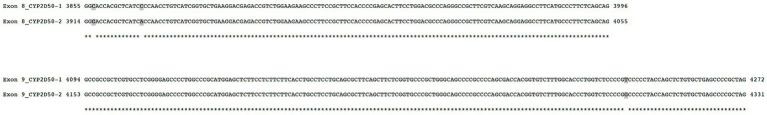
Alignment and comparison of exon 8 and exon 9 sequence between *CYP2D50–1* and *CYP2D50–2*. Positions are relative to genetic position, starting from ATG, for the two different genetic structures.

According to the EquCab3.0 assembly, *CYP2D50* (NC_009171.3) is represented in NCBI as an approximately 22Kb gene with 9 exons and a 17Kb long intron 7 (Figure 1A). Primer design revealed high sequence similarity between the region containing exons 1 to 7 and the sequence upstream of exon 8 (dashed area in Figure 1A); similarly, this was found also between the region containing exons 8 and 9 and the sequence downstream of exon 7 (dotted area in Figure 1A). This led to a deeper examination of sequence similarity within the gene, which revealed the presence of two putative genetic structures, both containing 9 exons separated by a 13Kb intergenic region (Figure 1B). The two putative genes are hereafter referred to as *CYP2D50–1* and *CYP2D50–2* and show nearly 95% identity at the coding sequence level (Table 1). Therefore, the structure currently depicted in NCBI would be a fusion between the first 7 exons of putative *CYP2D50–1* and the last two exons (8, 9) of putative *CYP2D50–2*. Exons 8 and 9 between the two putative genes differ by only 3 nucleotides, two in exon 8 and one in exon 9 (Figure 2).

**Table 1:** Percentage of identity between exons in the two putative genetic structures, *CYP2D50–1* and *CYP2D50–2*, defined by our model.

Exon	bps CYP2D50–1	bps CYP2D50–2	% of identity between 2D50-1 and 2D50-2
1	189	189	92
2	172	172	100
3	153	153	92
4	161	164	87
5	177	177	96
6	142	142	94
7	188	188	94
8	142	142	98
9	179	179	99

The percentage was calculated using MacVector Software. Bps: base pairs.

**Figure 3:**
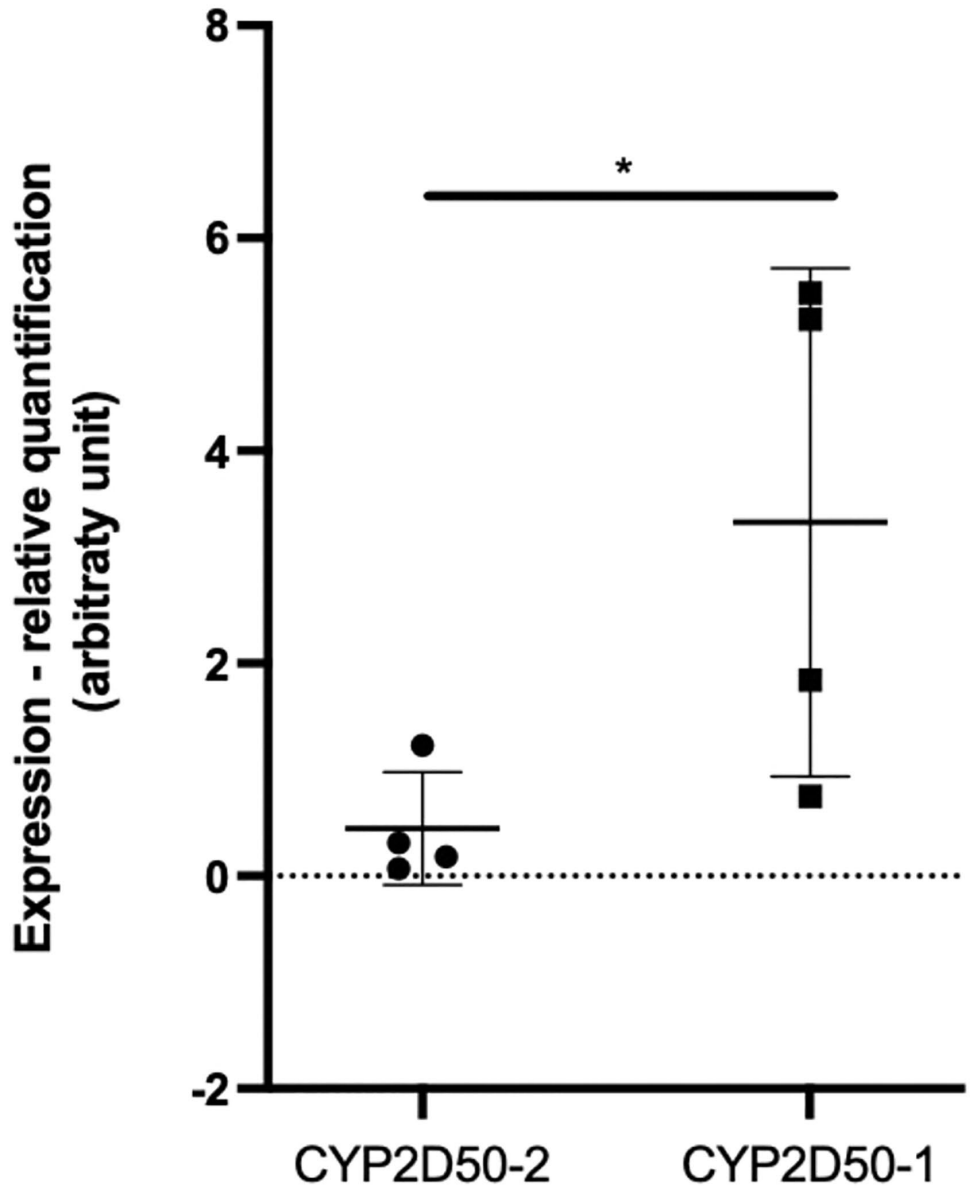
Real-Time PCR quantification of *CYP2D50–1* and *CYP2D50–2* transcripts on fresh horse liver mRNA extracted from 4 different horses. An asterisk (*) indicates a *p* value <0.05 after an unpaired t-test with Welch’s correction.

We first focused on *CYP2D50–1* because its genetic structure contains most of the exons defined for the *CYP2D50* gene in NCBI (7 of 9) and an alternative transcript for *CYP2D50* that resembles the structure of *CYP2D50–1* has already been proposed (XM_023631011.1). A PCR was designed to amplify all possible transcripts produced by *CYP2D50–1* and/or by the structure indicated in NCBI in mRNA extracts from fresh horse liver obtained from four different horses. For each of the four horses, the expected coding sequences (CDS) for *CYP2D50–1* and the NCBI genetic structure were reconstructed following genes amplification and sequencing from extracted gDNA. Alignment of the sequenced transcript of each horse with the corresponding CDS showed a complete match with the proposed *CYP2D50–1* gene and not with the NCBI structure (Table 2). The transcripts were also aligned with the corresponding *CYP2D50–2* CDS, and no match was found. A complete list of all mismatches found between the transcript and the *CYP2D50–2* CDS for each of the four horses can be found in Supplemental Tables S3–S6. Critical to the analysis of the results was the specific pattern of polymorphisms and resulting genotype identified in the amplified transcripts, particularly in the region of exon 8 and 9, which represents the only CDS difference between *CYP2D50–1* and the NCBI genetic structures. The polymorphisms identified in the transcript were completely replicated only in the *CYP2D50–1* gene with respect to each of the four sequenced horses (Table 2). A complete match to the corresponding genetic structure was also achieved by designing PCRs specific for *CYP2D50–1* and *CYP2D50–2* transcripts (data not shown). Since both *CYP2D50–1* and *CYP2D50–2* transcripts were detected, gene-specific qPCRs were developed to determine their relative expression in horse livers. The results showed that *CYP2D50–1* mRNA was expressed 7.5-fold more than *CYP2D50–2* (Figure 3). According to the overall results, the following sequences were submitted to the NCBI GenBank database: CYP2D50–1 mRNA, complete CDS (accession numbers OP596321, OP596322, OP596323, OP596324), CYP2D50–2 mRNA, complete CDS (accession number OP715798, requested CYP2D51 as new name for the gene).

**Table 2:** Polymorphisms identified in the four horses on the *CYP2D50* transcript.

	CYP2D50 transcript		CYP2D50–1 CDS	CYP2D50–2 CDS	CYP2D50-NCBI CDS
**Horse**	**Genetic coordinates from ATG**	**Genotype**	**Genotype**	**Genotype**	**Genotype**
#1 – EQU74	c.111	**C/A**	**HET (C/A)**	HOM (C/C)	HET (C/A)
	c.582	**C/T**	**HET (C/T)**	HOM (C/C)	HET (C/T)
	c.867	**G/T**	**HET (G/T)**	HOM (A/A)	HET (G/T)
	c.1185	**C/G**	**HET (C/G)**	HOM (G/G)	HOM (G/G)
	c.1198	**C/A**	**HET (C/A)**	HOM (A/A)	HOM (A/A)
	c.1471	**G/G**	**MUT (G/G)**	HOM (G/G)	HOM (G/G)
#2 – EQU75	c.517	**A/C**	**HET (A/C)**	HOM (C/C)	HET (A/C)
	c.522	**C/G**	**HET (C/G)**	HOM (G/G)	HET (C/G)
	c.582	**C/T**	**HET (C/T)**	HOM (C/C)	HET (C/T)
	c.867	**G/T**	**HET (G/T)**	HOM (A/A)	HET (G/T)
	c.1471	**T/G**	**HET (T/G)**	MUT (T/T)	MUT (T/T)
#3 – EQU76	c.111	**C/A**	**HET (C/A)**	HOM (C/C)	HET (C/A)
	c.582	**T/T**	**MUT (T/T)**	HOM (C/C)	MUT (T/T)
	c.867	**T/T**	**MUT (T/T)**	HOM (A/A)	MUT (T/T)
	c.1290	**C/T**	**HET (C/T)**	MUT (T/T)	MUT (T/T)
	c.1471	**G/G**	**MUT (G/G)**	HOM (G/G)	HOM (G/G)
#4 – EQU77	c.111	**C/A**	**HET (C/A)**	HOM (C/C)	HET (C/A)
	c.582	**C/T**	**HET (C/T)**	HOM (C/C)	HET (C/T)
	c.867	**G/T**	**HET (G/T)**	HOM (A/A)	HET (G/T)
	c.1185	**C/G**	**HET (C/G)**	HOM (G/G)	HOM (G/G)
	c.1198	**C/A**	**HET (C/A)**	HOM (A/A)	HOM (A/A)
	c.1471	**G/G**	**MUT (G/G)**	HET (G/T)	HET (G/T)

A comparison with the genotype for the corresponding positions of the reconstructed CDS for *CYP2D50–1*, *CYP2D50–2* and *CYP2D50* genes as depicted in NCBI is shown. A complete match is found only between the transcript and the *CYP2D50–1* CDS.

### The complexity of the CYP2D genes family

Several genes in the region around the *CYP2D50* location on chromosome 28 show a high degree of similarity to each other and to the *CYP2D50* gene at the genetic level (Table 3). For some of these genes, expression remains to be confirmed. PCR amplification identified full-length transcripts for LOC100146596 (*CYP2D84*), LOC100147480 (*CYP2D86*), and LOC100070990 (*CYP2D82*) in fresh liver tissue. LOC100070905 was also shown to be expressed, although the PCR used was not-target specific and cloning of the PCR product into vectors was necessary to screen the different amplicons obtained. On the other hand, LOC100056087 (*CYP2D89*) was shown not to be expressed using a series of primers targeting its specific transcript. LOC100070895 and LOC100070962 (*CYP2D88*) were also not expressed, although this conclusion was drawn indirectly. Indeed, the primers used to amplify CYP2D50–1 based on the genomic sequence of LOC100070895 and LOC100070962 would also have been able to amplify the transcript of these two genes, if present, resulting in three different sequences. The fact that such PCR always resulted in the specific amplification of only the CYP2D50–1 transcript proves that LOC100070895 and LOC100070962 are not expressed or at least not detectable.

**Table 3:** Percentage of identity between adjacent loci in the *CYP2D50* region at the genetic level (gDNA) from the ATG to the stop codon. 5’ UTR and 3’UTR regions were not considered in the analysis.

	CYP2D89 (LOC100056087)	CYP2D14 (LOC100070895)	CYP2D14 (LOC100070905)	CYP2D88 (LOC100070962)	CYP2D84 (LOC100146596)	CYP2D86 (LOC100147480)	CYP2D82 (LOC100070990)	CYP2D50–1	CYP2D50–2
CYP2D89 (LOC100056087)	x	82,9	78,7	83,5	83,8	81,5	81,8	83,7	83,2
CYP2D14 (LOC100070895)	–	x	86,5	85,1	85,1	80,1	80	95,1	93
CYP2D14 (LOC100070905)	–	–	x	80,7	80,2	78,8	78,4	86,7	86,5
CYP2D88 (LOC100070962)	–	–	–	x	84,2	79,9	79,8	85,4	84,6
CYP2D84 (LOC100146596)	–	–	–	–	x	81,0	81,2	85,7	85,4
CYP2D86 (LOC100147480)	–	–	–	–	–	x	94,9	80,8	80,8
CYP2D82 (LOC100070990)	–	–	–	–	–	–	x	80,4	80,6
CYP2D50–1	–	–	–	–	–	–	–	x	93,7
CYP2D50–2	–	–	–	–	–	–	–	–	x

Percentage was calculated using MacVector Software.

### Sequencing of CYP2D50–1 and CYP2D82 genes revealed several polymorphisms

Of the members of the *CYP2D* family in horses, only *CYP2D50* and *CYP2D82* have been shown to be involved in drug metabolism. To determine the possible SNPs that might affect their function, gDNA corresponding to *CYP2D50–1* and *CYP2D82* was sequenced from all 72 horses in our cohort.

For the *CYP2D50–1* gene, 54 mutations were identified, 23 of which were in exons, including 10 that caused an amino acid change (Supplemental Table S7). Most of these mutations are already associated with an rs number in Ensembl, but 16 of them were observed for the first time. Of the newly identified mutations, 6 cause an amino acid change at positions R31H, G45E, G376A, T486I, L487V, and S489F. All 6 substitutions were tested for functional impact using predictive tools such as SIFT and PROVEAN, and two of them, R31H and G45E, were predicted to be deleterious mutations that severely impair protein function (Table 4).

**Table 4:** List of mutations altering an amino acid found in the *CYP2D50–1* and *CYP2D82* genes with the resulting score for effects on enzymatic activity derived from PROVEAN and SIFT.

CYP2D50–1						
**Location (CYP2D50–1 from ATG)**	**SNP identified**	**Aminoacidic change**	**Frequency alleles containing the SNP (%)**	**rs number**	**PROVEAN result**	**SIFT results**
g.92	G > A	R31H	3.47		**−3.134**	**0.05**
g.134	G > A	G45E	0.69		**−3.972**	**0.02**
g.2605	C > A	Q269K	1.39	rs1148503187	**−3.289**	**0.05**
g.3328	G > C	G376A	0.69		**−3.397**	0.25
g.4192	C > A	Q475K	1.39	rs1137288236	**−3.149**	0.09
g.4226	C > T	T486I	7.64		2.112	0.36
g.4228	C > G	L487V	7.64		−1.340	0.35
g.4235	C > T	S489F	0.69		−2.281	0.11
g.4240	T > G	S491A	42.36	rs396861920	−0.461	0.65
g.4255	T > A	C496S	7.64	rs394932323	**−8.369**	**0**
**CYP2D82**						
**Location (NC_009171.3 from ATG)**	**SNP identified**	**Aminoacidic change**	**Frequency alleles containing the SNP (%)**	**rs number**	**PROVEAN result**	**SIFT results**
g.83	G > A	R28Q	18.06	rs1141439398	**−3.327**	0.09
g.85	C > T	R29C	0.69	rs1150936359	**−6.934**	**0**
g.1888	C > G	R126G	12.50		**−2.807**	0.20
g.1892	A > C	Y127S	1.39		**−6.190**	0.22
g.1984	G > C	E158Q	5.56	rs1143153764	−1.719	0.27
g.2214	T > G	L204R	5.56	rs1138659802	2.658	0.65
g.2628	C > T	L233F	1.39		**−2.615**	**0.03**
g.2635	A > G	H235R	1.39	rs1147081710	−1.112	1
g.2646	G > C	V239L	0.69		1.753	1
g.2650	C > T	A240V	0.69		−1.128	1
g.2673	A > G	K248E	5.56	rs1145722969	−2.428	0.21
g.2674	A > G	K248R	54.86	rs1143545655	−0.972	0.20
g.2673–2,675	AAG > GGA	K248G	0.72		**−4.673**	**0.04**
g.2691	C > G	L254V	68.06	rs394128659	−1.482	0.72
g.2703	C > G	L258V	34.03		−0.395	0.55
g.2733	A > G	T268A	1.39	rs1136055998	1.527	1
g.2767	T > A	L279Q	1.39	rs1142815816	**−5.151**	**0**
g.3403	T > C	C355R	68.06	rs396631299	4.494	0.74
g.3469	T > G	L377V	9.72	rs1148651933	0.477	1
g.3472	A > G	T378A	9.72	rs1136958956	−0.329	0.46
g.3474–3,484	CCACATGACAT del	378 fs	0.69			
g.3493	A > G	I385V	68.75	rs395645145	−0.035	0.17
g.4104	C > T	R417C	22.22	rs1148309512	**−4.238**	**0.02**
g.4141	G > A	R429H	5.56	rs1145664752	−0.548	0.61

Detrimental mutations likely to affect protein function are all variants with a score of −2.5 or less for PROVEAN and all variants with a score between 0 and 0.05 for SIFT. Other scores are indicative of tolerated mutations. Deleterious scores are indicated in bold.

Some of the SNPs identified are very common in the horses tested. 12 mutations were identified in more than 50% of the animals, and 6 of them were frequently detected in homozygosity. One SNP, g.1096 T > C in intron 2, occur in homozygosity in all horses. Although all of these mutations are located in introns or are synonymous variants, an exception is g.4240 T > G, which causes the amino acid mutation S491A and results in complete identity of exon 9 to the sequence of *CYP2D50–2*.

For the *CYP2D82* gene, 88 mutations were identified, 38 of which were in exons, of which 23 caused an amino acid change (Supplemental Table S8). Of the 40 SNPs that were not yet assigned an rs number in Ensembl, 6 cause an amino acid change at positions R126G, Y127S, L233F, V239L, A240V, and L258V. By analysis with SIFT and PROVEAN, only L233F was clearly defined as deleterious, whereas R126G and Y127S appeared as deleterious only with the PROVEAN tool (Table 4). Another newly identified mutation that adversely affects protein function is CCACATGACATdel at position g.3474-3484, resulting in a frameshift (378 fs).

### Definition of a CYP2D50–1 hybrid genetic structure

**Figure 4:**
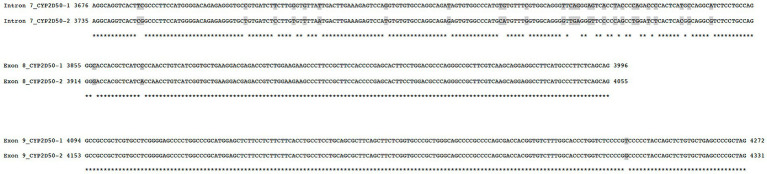
Comparison between the genomic sequence of *CYP2D50–1* and *CYP2D50–2* in parts of intron 7, exon 8, and exon 9. The sequence of intron 8 sequence is not shown because it is identical between the two genes. Positions are relative to the genetic position, starting with ATG for the two different genetic structures.

One variation observed in the genetic analysis of *CYP2D50* is a *CYP2D50–1* hybrid sequence determined by the conversion of exons 8 and 9 with *CYP2D50–2*. The switching region was located in intron 7 in the area between positions g.3625 and g.3687 on *CYP2D50–1*, and the hybrid structure was confirmed by the identification of several mismatches in the intron 7 region that perfectly reconstitute the *CYP2D50–2* sequence (Figure 4; Table 5). Among the mismatches that convert the *CYP2D50–1* sequence to a *CYP2D50–2* sequence, nucleotide C in position g.3805 in the *CYP250-1* sequence was never found converted to a T, as it should have been for *CYP2D50–2*. Sequencing of the *CYP2D50–2* gene from 9 horses in our cohort revealed a SNP present in homozygosity in all sequenced horses, mutating the T in position g.3864 to a C. Position g.3864 on the *CYP2D50–2* gene corresponds to position g.3805 on the *CYP2D50–1* gene. The presence of this T > C SNP in the *CYP2D50–2* gene explains why the corresponding position on the *CYP2D50–1* gene is never found converted in the *CYP2D50–1* hybrid structure. The *CYP2D50–1* hybrid sequence was identified in 29.17% of the alleles analyzed.

**Table 5:** List of all SNPs found in the *CYP2D50–1* gene between intron 7 and exon 9 converting the sequence to a perfect match with the *CYP2D50–2* gene, proving a gene conversion event.

Location (on gDNA CYP2D50–1)	SNP identified	Nucleotide on corresponding CYP2D50–2 location
g.3688	T > C	C
g.3689	C > G	G
g.3717	C > T	T
g.3725	T > C	C
g.3730	G > T	T
g.3733	G > T	T
g.3737	T > A	A
g.3755	G > T	T
g.3772	T > G	G
g.3786	T > C	C
g.3787	G > A	A
g.3793	C > G	G
g.3803	T > G	G
g.3805	C > T	T
g.3806	A > G	G
g.3807	G > A	A
g.3810	A > G	G
g.3811	G > T	T
g.3814	A > C	C
g.3817	T > C	C
g.3819	C > G	G
g.3822	C > T	T
g.3823	A > G	G
g.3826	C > T	T
g.3828	C > T	T
g.3835	T > C	C
g.3837	C > G	G
g.3843	A > G	G
g.3857	C ≥ G	G
g.3870	C ≥ A	A
g.4240	T ≥ G	G

The underlined genomic positions are in exons 8 and 9, and all other mutations are in intron 7. The grey highlighted position g.3805 is a position that is frequently mutated in the *CYP2D50–2* gene.

## Discussion

Although equine pharmacogenetics may play an increasingly important role in safer therapeutic treatment and appropriate drug withdrawal (20), the involvement of equine metabolic enzymes has not yet been extensively studied. In this view, we analyzed the genetics of the equine orthologue family for *CYP2D6*. Our initial focus was on *CYP2D50*, which had a higher level of complexity than previously known. Two very similar genes were identified in the genetic structure previously assigned to the single *CYP2D50* gene in NCBI. These two genes, designated as *CYP2D50–1* and *CYP2D50–2*, are both expressed in liver tissue from 4 different horses, although *CYP2D50–1* is 7.5-fold more expressed than *CYP2D50–2*. Overall, the data suggest that the genetic structure of *CYP2D50* defined in the latest NCBI EquCab3.0 assembly is likely a fusion of two different genes, *CYP2D50–1* and *CYP2D50–2*, and the former is the most abundant of the two transcripts in horse liver. *CYP2D50* is the first gene thought to be the orthologous gene of *CYP2D6* in the horse. The *CYP2D6* gene locus in humans contains three genes, *CYP2D6, CYP2D7,* and *CYP2D8,* the latter two of which are considered pseudogenes and have no functional relevance. However, they are very similar to *CYP2D6* at the sequence level and this should always be taken into account in genetic analysis and assay design based on primer and/or probe binding. The two genes *CYP2D50–1* and *CYP2D50–2* may resemble the gene locus structure in humans, with one being expressed and enzymatically functional and the second being a pseudogene. From the literature, *CYP2D50–1* is most likely a functional gene (28), whereas further studies are needed for *CYP2D50–2*. For specific PCR design, sequence identity between these two genes must be considered, as well as identity with the other 7 genes previously identified in horses that belong to the CYP2D family, 4 of which have been shown to be expressed at the mRNA level in horse liver. For one of them, *CYP2D82*, expression and function have already been defined (29, 39), whereas for the other 3 (*CYP2D84*, *CYP2D86,* and *CYP2D14*-LOC100070905) no evidence for functionality is available and their expression in other tissues is also unknown. Considering that CYP2D50 and CYP2D82 are involved in the metabolism of two different substrates that are metabolized by CYP2D6 in humans, a study of the target specificity or redundancy of metabolic pathways for the different enzymes would be necessary to identify genes relevant to specific pharmacological treatments if other CYP2D genes are functionally active in horse. Understanding gene locus structure, expression patterns, and enzyme functionality is an essential step toward deeper pharmacogenetic analysis.

An in-depth study of genetic variations was developed only for the two genes for which a function has already been defined, namely *CYP2D50–1* and *CYP2D82*. Several new polymorphisms have been discovered for both genes, some of which determine an amino acid change. In a study with the *in-silico* prediction tools SIFT and PROVEAN, only 2 of these mutations (R31H and G45E) in *CYP2D50–1* and one (L233F) in *CYP2D82* were clearly identified as deleterious. These mutations were found in heterozygosity in 5 horses for R31H, in 1 horse for G45E, and in 2 horses for L233F, and if the predictions are correct, it would not be surprising to find metabolic impairment for some drugs in these animals. However, the effect of polymorphisms on the function of an enzyme could only be specifically analyzed if a functional assay was developed, and unfortunately this was not possible in the current study.

Interestingly, analysis of *CYP2D50–1* also revealed the possibility of a genetic conversion, a well-known phenomenon that has been described in detail for *CYP2D6* in humans (44). An exon 9 conversion and various hybrid structures can be formed between *CYP2D6* and *CYP2D7,* and the results of the genetic analyzes in this study indicate that they are formed similarly between *CYP2D50–*1 and *CYP2D50–2* in the horse genome. Considering that all human *CYP2D6::CYP2D7* hybrids are known to be nonfunctional, we might expect the same to be true for *CYP2D50–1* and *CYP2D50–2* hybrids, although this assumption could only be confirmed experimentally with a functional assay. Since multiplication and deletion of alleles are two other known phenomena for *CYP2D6* in humans and can similarly occur in horses (45), the development of specific copy number variation (CNV) assays would be required to overcome some limitations (such as the use of complex algorithms, signal normalization, high background noise, and high error rate in regions of low SNP density (46)) and complement assays based only on SNP to correctly define the equine genotype.

The road to pharmacogenetic analysis in horses is still long because of the lack of knowledge about these genes, their function, and the effects of polymorphisms on them. This study allowed us to gain new insights into the genetic structure of *CYP2D50* by identifying two new genes (*CYP2D50–1* and *CYP2D50–2*) and describing the presence of hybrid structures similar to those observed in humans between *CYP2D6* and *CYP2D7*. Extensive sequencing also allowed the identification of novel polymorphisms in *CYP250-1* and *CYP2D82* also associated with amino acid changes, whose impact would only be defined with a dedicated functional test. In addition, we have begun to unravel the complex structure of the CYP2D family in the horse, highlighting the high sequence similarity between these genes and the associated difficulties for the design of specific PCRs.

## Data availability statement

The datasets presented in this study can be found in online repositories. The names of the repository/repositories and accession number(s) can be found in the article/Supplementary material.

## Ethics statement

Ethical approval was not required for the studies involving animals in accordance with the local legislation and institutional requirements because venous blood from horses was collected for clinical (rather than scientific) purposes at a veterinary hospital, and the excess sample was donated to PharmGenetix. Liver tissue from horses in the study was obtained from animals that were not euthanized for scientific purposes and donated to PharmGenetix by a veterinary clinic. Written informed consent was not obtained from the owners for the participation of their animals in this study because venous blood from horses was collected for clinical (rather than scientific) purposes at a veterinary hospital, and the excess sample was donated to PharmGenetix. Liver tissue from horses in the study was obtained from animals that were not euthanized for scientific purposes and donated to PharmGenetix by a veterinary clinic.

## Author contributions

GS, SV, and CN participated in the study conceptualization and experimental design. GS and SV drafted the manuscript. GS performed the experiments. GS, SV, MP, and CN participated in data analysis and/or interpretation. All authors contributed to the article and approved the submitted version.
