# Supplementary material for: Genetic analysis of the equine orthologues for human CYP2D6: unraveling the complexity of the CYP2D family in horses
Source: Front Vet Sci. 2023 Oct 19;10:1188633. doi: 10.3389/fvets.2023.1188633 (PMC10620600; doi:10.3389/fvets.2023.1188633)
Supplement: Supplementary file 1 [file Data_Sheet_1.docx]

Genetic analysis of the equine orthologs for human CYP2D6: unraveling the complexity of the CYP2D family in horses – Supplemental tables

Giada Scantamburlo^1*^, Simone Vanoni^1^, Markus Paulmichl^2^ and Charity Nofziger^1^

^1^ Pharmgenetix GmbH, Sonystrasse 20, 5081 Anif, Austria

^2^ Privatklinik Maria Hilf GmbH, Radetzkystrasse 35, 9020 Klagenfurt, Austria

**Supplemental table 1: End-point and Real-Time PCR primers and parameters.** ACTB: b-actin; bps: base pairs; fwd: forward; rev: reverse; Tm: melting temperature.

| Reaction | Tm (°C) | Primer fwd (5‘>3‘) | Primer rev (5‘>3‘) | Amplicon length (bps) |
| --- | --- | --- | --- | --- |
| **End-point PCR** | | | | |
| CYP2D50-1 gene | 68,3 | AGTGTGCACTGAGGCAGCCATGG | GGGGCAAGCTGTCACCTTCTGAGTC | 4532 |
| CYP2D50-1 transcript | 54 | AGTGTGCACTGAGGCAGCCATGG | GGGGCAAGCTGTCACCTTCTGAGTC | 1763 |
| CYP2D50-2 gene | 68,3 | AGTGTGCACTGAGGCAGCCATGG | ACCCTGTCACAAGGCTGTATAGGCG | 4538 |
| CYP2D50-2 transcript | 68,3 | AGTGTGCACTGAGGCAGCCATGG | ACCCTGTCACAAGGCTGTATAGGCG | 1503 |
| CYP2D50 general transcript | 54 | ATGGGGCTGCTGACCTGG | CTAGCGGGGCTCAGC | 1503 |
| CYP2D82 gene | 58,4 | GAGGAGCGCAGTGTACAC | GCATCTCTGCTCAGGGG | 4597 |
| CYP2D82 transcript | 58,4 | GAGGAGCGCAGTGTACAC | GCATCTCTGCTCAGGGG | 1503 |
| LOC100056087 (CYP2D89) transcript | 57,3 | CACCGAGGCAACCATGG | GCCAGGTTGGAGCATCATAC | 1591 |
| LOC100146596 CYP2D86 transcript | 61,4 | AGTGTGCACTGAGGCAGCCATGG | GGGGTCATTATGGAGCGAAAG | 1488 |
| LOC100147480 CYP2D84 transcript | 68,3 | AGTGTGCACTGAGGCAGCCATGG | CACGGTTGGGGGCTTGGTGCAC | 1547 |
| LOC100070905 transcript | 58 | AGTGTGCACTGAGGCAACCATGG | CAAGGAGGGGTAGGATCTGTCC | 1687 |
| **Real-Time PCR** | | | | |
| ACTB | 55 | ﻿AGGGAAATCGTGCGTGACAT | ﻿CTCGTTGCCGATGGTGATGA | 144 |
| CYP2D50-1 | 55 | ﻿GCGGAAGGCCCTTTAGC | ﻿CAGTATGTCCTCTGTTAGGTCCAATATC | 137 |
| CYP2D50-2 | 55 | ﻿GAACCCGCACTGCTGC | ﻿CCACGTTGGGCAAAGATCAC | 219 |

**Supplemental table 2: Sequencing primers.**

| Primer ID | Primer sequence (5‘>3‘) |
| --- | --- |
| 211 | ATGGGGCTGCTGACCTGG |
| 212 | CTAGCGGGGCTCAGC |
| 220 | CCGCTTTATTCAGGAGGG |
| 221 | CGATCTCCTGTTGGACAC |
| 222 | GAAGGCCCTTTAGCCCCGAC |
| 223 | CATGATCCTGCACCCGGATG |
| 228 | AGTGTGCACTGAGGCAGCCATGG |
| 231 | CTGCTCAGGGGTCTCAGGTGG |
| 265 | CAAGACTAGAGCCAGGAGACT |
| 266 | GGGTGGGCTAAGCTACCATC |
| 267 | CACTGAAGGAGGTAGAGGCTC |
| 253 | ACCCTGTCACAAGGCTGTATAGGCG |
| 254 | GGGGCAAGCTGTCACCTTCTGAGTC |
| 331 | GGTGTCCTCGCCGCGGTGC |
| 395 | GAGGAGCGCAGTGTACAC |
| 396 | GCATCTCTGCTCAGGGG |
| 397 | TTCGAGTACAACGACCCGC |
| 398 | CCGGGTGCAGGATCATGA |
| 507 | CAGGGAGGTGAGCAGGGAGGC |
| 508 | CCGCCTTCCAGTTCAAG |
| 509 | CGCCCTCCTTGTCGTCCTCC |
| 510 | CTTCCTGGCACCCTCCCTGC |
| 511 | CCAATTTCCCCGTCTGGC |
| 512 | GATGGTGTAAAGTGGGCAGG |
| 513 | GATGGGTGCAGAGTGGG |
| 514 | GCCCCATCGTGTTGCAG |

**Supplemental Table 3:** **Complete list of the mismatches found between CYP2D50 transcript and reconstructed CYP2D50-2 CDS for horse EQU74.**

| EQU74 | | | | |
| --- | --- | --- | --- | --- |
|  | **CYP2D50 transcript** | | **CYP2D50-2 gene** | |
| Exon | **Genetic coordinates from ATG** | **Genotype** | **Reference nucleotide** | **Genotype** |
| 1 | c.16 | **T/T** | G | G/G |
| 1 | c.21 | **T/T** | G | G/G |
| 1 | c.23 | **A/A** | C | C/C |
| 1 | c.31 | **C/C** | A | A/A |
| 1 | c.33 | **T/T** | G | G/G |
| 1 | c.34 | **G/G** | C | C/C |
| 1 | c.39 | **T/T** | C | C/C |
| 1 | c.111 | **C/A** | C | C/C |
| 1 | c.166 | **G/G** | A | A/A |
| 1 | c.169 | **A/A** | C | C/C |
| 1 | c.172 | **G/G** | C | C/C |
| 1 | c.173 | **T/T** | A | A/A |
| 1 | c.174 | **T/T** | C | C/C |
| 1 | c.176 | **C/C** | G | G/G |
| 1 | c.178 | **A/A** | T | T/T |
| 1 | c.188 | **G/G** | C | C/C |
| 3 | c.367 | **G/G** | A | A/A |
| 3 | c.377 | **G/G** | A | A/A |
| 3 | c.378 | **C/C** | A | A/A |
| 3 | c.379 | **T/T** | C | C/C |
| 3 | c.380 | **A/A** | G | G/G |
| 3 | c.389 | **C/C** | T | T/T |
| 3 | c.435 | **C/C** | T | T/T |
| 3 | c.439 | **C/C** | A | A/A |
| 3 | c.472 | **C/C** | G | G/G |
| 3 | c.485 | **A/A** | G | G/G |
| 3 | c.497 | **T/T** | C | C/C |
| 3 | c.512 | **G/G** | C | C/C |
| 4 | c.517 | **A/A** | C | A/A |
| 4 | c.522 | **C/C** | G | C/C |
| 4 | c.532 | **G/G** | A | G/G |
| 4 | c.582 | **C/T** | C | C/C |
| 4 | c.594 | **T/T** | C | T/T |
| 4 | c.597 | **C/C** | G | C/C |
| 4 | c.601-603 |  | AAC | del |
| 4 | c.610 | **C/C** | T | C/C |
| 4 | c.611 | **A/A** | G | A/A |
| 4 | c.618 | **T/T** | C | T/T |
| 4 | c.619 | **G/G** | A | G/G |
| 4 | c.635 | **C/C** | T | T/T |
| 4 | c.636 | **A/A** | G | G/G |
| 4 | c.641 | **A/A** | G | G/G |
| 4 | c.643 | **A/A** | T | T/T |
| 4 | c.645 | **A/A** | T | T/T |
| 4 | c.655 | **C/C** | G | G/G |
| 4 | c.656 | **A/A** | C | C/C |
| 4 | c.667 | **C/C** | G | G/G |
| 5 | c.683 | **A/A** | G | G/G |
| 5 | c.684 | **C/C** | T | T/T |
| 5 | c.694 | **A/A** | G | G/G |
| 5 | c.782 | **G/G** | A | A/A |
| 5 | c.813 | **T/T** | C | C/C |
| 5 | c.828 | **T/T** | C | C/C |
| 5 | c.836 | **T/T** | A | A/A |
| 6 | c.867 | **G/T** | A | A/A |
| 6 | c.919 | **G/G** | T | T/T |
| 6 | c.920 | **C/C** | T | T/T |
| 6 | c.921 | **C/C** | T | T/T |
| 6 | c.924 | **T/T** | C | C/C |
| 6 | c.942 | **A/A** | G | G/G |
| 6 | c.945 | **A/A** | C | C/C |
| 6 | c.946 | **G/G** | A | A/A |
| 7 | c.1012 | **A/A** | G | G/G |
| 7 | c.1064 | **G/G** | A | A/A |
| 7 | c.1109 | **G/G** | C | C/C |
| 7 | c.1117 | **G/G** | A | A/A |
| 7 | c.1123 | **G/G** | C | C/C |
| 7 | c.1128 | **C/C** | T | T/T |
| 7 | c.1130 | **C/C** | T | T/T |
| 7 | c.1131 | **G/G** | A | A/A |
| 7 | c.1143 | **G/G** | A | A/A |
| 7 | c.1176 | **T/T** | C | C/T |
| 7 | c.1181 | **A/A** | G | G/G |
| 8 | c.1185 | **C/G** | G | G/G |
| 8 | c.1198 | **C/A** | A | A/A |
| 9 | c.1471 | **G/G** | G | G/G |

**Supplemental Table 4:** **Complete list of the mismatches found between CYP2D50 transcript and reconstructed CYP2D50-2 CDS for horse EQU75.**

| EQU75 | | | | |
| --- | --- | --- | --- | --- |
|  | **CYP2D50 transcript** | | **CYP2D50-2 gene** | |
| Exon | **Genetic coordinates from ATG** | **Genotype** | **Reference nucleotide** | **Genotype** |
| 1 | c.16 | **T/T** | G | G/G |
| 1 | c.21 | **T/T** | G | G/G |
| 1 | c.23 | **A/A** | C | C/C |
| 1 | c.31 | **C/C** | A | A/A |
| 1 | c.33 | **T/T** | G | G/G |
| 1 | c.34 | **G/G** | C | C/C |
| 1 | c.39 | **T/T** | C | C/C |
| 1 | c.73 | **C/C** | C | G/G |
| 1 | c.102 | **A/A** | A | C/C |
| 1 | c.166 | **G/G** | A | A/A |
| 1 | c.169 | **A/A** | C | C/C |
| 1 | c.172 | **G/G** | C | C/C |
| 1 | c.173 | **T/T** | A | A/A |
| 1 | c.174 | **T/T** | C | C/C |
| 1 | c.176 | **C/C** | G | G/G |
| 1 | c.178 | **A/A** | T | T/T |
| 1 | c.188 | **G/G** | C | C/C |
| 3 | c.367 | **G/G** | A | A/A |
| 3 | c.377 | **G/G** | A | A/A |
| 3 | c.378 | **C/C** | A | A/A |
| 3 | c.379 | **T/T** | C | C/C |
| 3 | c.380 | **A/A** | G | G/G |
| 3 | c.389 | **C/C** | T | T/T |
| 3 | c.435 | **C/C** | T | T/T |
| 3 | c.439 | **C/C** | A | A/A |
| 3 | c.472 | **C/C** | G | G/G |
| 3 | c.485 | **A/A** | G | G/G |
| 3 | c.497 | **T/T** | C | C/C |
| 3 | c.512 | **G/G** | C | C/C |
| 4 | c.517 | **A/C** | C | C/C |
| 4 | c.522 | **C/G** | G | G/G |
| 4 | c.532 | **G/G** | A | A/A |
| 4 | c.567 | **C/C** | C | G/G |
| 4 | c.582 | **C/T** | C | C/C |
| 4 | c.594 | **T/T** | C | C/C |
| 4 | c.597 | **C/C** | G | G/G |
| 4 | c.601-603 |  | AAC | AAC |
| 4 | c.610 | **C/C** | T | T/T |
| 4 | c.611 | **A/A** | G | G/G |
| 4 | c.618 | **T/T** | C | C/C |
| 4 | c.619 | **G/G** | A | A/A |
| 4 | c.635 | **C/C** | T | T/T |
| 4 | c.636 | **A/A** | G | G/G |
| 4 | c.641 | **A/A** | G | G/G |
| 4 | c.643 | **A/A** | T | T/T |
| 4 | c.645 | **A/A** | T | T/T |
| 4 | c.655 | **C/C** | G | G/G |
| 4 | c.656 | **A/A** | C | C/C |
| 4 | c.667 | **C/C** | G | G/G |
| 5 | c.683 | **A/A** | G | G/G |
| 5 | c.684 | **C/C** | T | T/T |
| 5 | c.694 | **A/A** | G | G/G |
| 5 | c.782 | **G/G** | A | A/A |
| 5 | c.813 | **T/T** | C | C/C |
| 5 | c.828 | **T/T** | C | C/C |
| 5 | c.836 | **T/T** | A | A/A |
| 6 | c.867 | **G/T** | A | A/A |
| 6 | c.919 | **G/G** | T | T/T |
| 6 | c.920 | **C/C** | T | T/T |
| 6 | c.921 | **C/C** | T | T/T |
| 6 | c.924 | **T/T** | C | C/C |
| 6 | c.930 | **G/G** | G | A/A |
| 6 | c.942 | **A/A** | G | G/G |
| 6 | c.945 | **A/A** | C | C/C |
| 6 | c.946 | **G/G** | A | A/A |
| 7 | c.1012 | **A/A** | G | G/G |
| 7 | c.1064 | **G/G** | A | A/A |
| 7 | c.1109 | **G/G** | C | C/C |
| 7 | c.1117 | **G/G** | A | A/A |
| 7 | c.1123 | **G/G** | C | C/C |
| 7 | c.1128 | **C/C** | T | T/T |
| 7 | c.1130 | **C/C** | T | T/T |
| 7 | c.1131 | **G/G** | A | A/A |
| 7 | c.1143 | **G/G** | A | A/A |
| 7 | c.1176 | **T/T** | C | C/C |
| 7 | c.1181 | **A/A** | G | G/G |
| 8 | c.1185 | **C/C** | G | G/G |
| 8 | c.1198 | **C/C** | A | A/A |
| 8 | c.1276 | **G/G** | G | A/A |
| 9 | c.1457 | **C/C** | C | T/T |
| 9 | c.1459 | **C/C** | C | G/G |
| 9 | c.1470 | **G/G** | G | A/A |
| 9 | c.1471 | **T/G** | G | T/T |
| 9 | c.1485 | **C/C** | C | T/T |
| 9 | c.1486 | **T/T** | T | A/A |

**Supplemental Table 5:** **Complete list of the mismatches found between CYP2D50 transcript and reconstructed CYP2D50-2 CDS for horse EQU76.**

| EQU76 | | | | |
| --- | --- | --- | --- | --- |
|  | **CYP2D50 transcript** | | **CYP2D50-2 gene** | |
| Exon | **Genetic coordinates from ATG** | **Genotype** | **Reference nucleotide** | **Genotype** |
| 1 | c.16 | **T/T** | G | G/G |
| 1 | c.21 | **T/T** | G | G/G |
| 1 | c.23 | **A/A** | C | C/C |
| 1 | c.31 | **C/C** | A | A/A |
| 1 | c.33 | **T/T** | G | G/G |
| 1 | c.34 | **G/G** | C | C/C |
| 1 | c.39 | **T/T** | C | C/C |
| 1 | c.111 | **C/A** | C | C/C |
| 1 | c.166 | **G/G** | A | A/A |
| 1 | c.169 | **A/A** | C | C/C |
| 1 | c.172 | **G/G** | C | C/C |
| 1 | c.173 | **T/T** | A | A/A |
| 1 | c.174 | **T/T** | C | C/C |
| 1 | c.176 | **C/C** | G | G/G |
| 1 | c.178 | **A/A** | T | T/T |
| 1 | c.188 | **G/G** | C | C/C |
| 3 | c.367 | **G/G** | A | A/A |
| 3 | c.377 | **G/G** | A | A/A |
| 3 | c.378 | **C/C** | A | A/A |
| 3 | c.379 | **T/T** | C | C/C |
| 3 | c.380 | **A/A** | G | G/G |
| 3 | c.389 | **C/C** | T | T/T |
| 3 | c.435 | **C/C** | T | T/T |
| 3 | c.439 | **C/C** | A | A/A |
| 3 | c.472 | **C/C** | G | G/G |
| 3 | c.485 | **A/A** | G | G/G |
| 3 | c.497 | **T/T** | C | C/C |
| 3 | c.512 | **G/G** | C | C/C |
| 4 | c.517 | **A/A** | C | A/A |
| 4 | c.522 | **C/C** | G | C/C |
| 4 | c.532 | **G/G** | A | G/G |
| 4 | c.582 | **T/T** | C | C/C |
| 4 | c.594 | **T/T** | C | T/T |
| 4 | c.597 | **C/C** | G | C/C |
| 4 | c.601-603 |  | AAC | del |
| 4 | c.610 | **C/C** | T | C/C |
| 4 | c.611 | **A/A** | G | A/A |
| 4 | c.618 | **T/T** | C | T/T |
| 4 | c.619 | **G/G** | A | G/G |
| 4 | c.635 | **C/C** | T | T/T |
| 4 | c.636 | **A/A** | G | G/G |
| 4 | c.641 | **A/A** | G | G/G |
| 4 | c.643 | **A/A** | T | T/T |
| 4 | c.645 | **A/A** | T | T/T |
| 4 | c.655 | **C/C** | G | G/G |
| 4 | c.656 | **A/A** | C | C/C |
| 4 | c.667 | **C/C** | G | G/G |
| 5 | c.683 | **A/A** | G | G/G |
| 5 | c.684 | **C/C** | T | T/T |
| 5 | c.694 | **A/A** | G | G/G |
| 5 | c.782 | **G/G** | A | A/A |
| 5 | c.813 | **T/T** | C | T/T |
| 5 | c.828 | **T/T** | C | C/C |
| 5 | c.836 | **T/T** | A | A/A |
| 6 | c.867 | **T/T** | A | A/A |
| 6 | c.919 | **G/G** | T | T/T |
| 6 | c.920 | **C/C** | T | T/T |
| 6 | c.921 | **C/C** | T | T/T |
| 6 | c.924 | **T/T** | C | C/C |
| 6 | c.942 | **A/A** | G | G/G |
| 6 | c.945 | **A/A** | C | C/C |
| 6 | c.946 | **G/G** | A | A/A |
| 7 | c.1012 | **A/A** | G | G/G |
| 7 | c.1064 | **G/G** | A | A/A |
| 7 | c.1092 | **C/C** | C | C/T |
| 7 | c.1109 | **G/G** | C | C/C |
| 7 | c.1117 | **G/G** | A | A/A |
| 7 | c.1123 | **G/G** | C | C/C |
| 7 | c.1128 | **C/C** | T | T/T |
| 7 | c.1130 | **C/C** | T | T/T |
| 7 | c.1131 | **G/G** | A | A/A |
| 7 | c.1143 | **G/G** | A | A/A |
| 7 | c.1176 | **T/T** | C | C/T |
| 7 | c.1181 | **A/A** | G | G/G |
| 8 | c.1185 | **C/C** | G | G/G |
| 8 | c.1198 | **C/C** | A | A/A |
| 8 | c.1290 | **C/T** | C | T/T |
| 9 | c.1471 | **G/G** | G | G/G |

**Supplemental Table 6:** **Complete list of the mismatches found between CYP2D50 transcript and reconstructed CYP2D50-2 CDS for horse EQU77.**

| EQU77 | | | | |
| --- | --- | --- | --- | --- |
|  | **CYP2D50 transcript** | | **CYP2D50-2 gene** | |
| Exon | **Genetic coordinates from ATG** | **Genotype** | **Reference nucleotide** | **Genotype** |
| 1 | c.16 | **T/T** | G | G/G |
| 1 | c.21 | **T/T** | G | G/G |
| 1 | c.23 | **A/A** | C | C/C |
| 1 | c.31 | **C/C** | A | A/A |
| 1 | c.33 | **T/T** | G | G/G |
| 1 | c.34 | **G/G** | C | C/C |
| 1 | c.39 | **T/T** | C | C/C |
| 1 | c.111 | **C/A** | C | C/C |
| 1 | c.166 | **G/G** | A | A/A |
| 1 | c.169 | **A/A** | C | C/C |
| 1 | c.172 | **G/G** | C | C/C |
| 1 | c.173 | **T/T** | A | A/A |
| 1 | c.174 | **T/T** | C | C/C |
| 1 | c.176 | **C/C** | G | G/G |
| 1 | c.178 | **A/A** | T | T/T |
| 1 | c.188 | **G/G** | C | C/C |
| 3 | c.367 | **G/G** | A | A/A |
| 3 | c.377 | **G/G** | A | A/A |
| 3 | c.378 | **C/C** | A | A/A |
| 3 | c.379 | **T/T** | C | C/C |
| 3 | c.380 | **A/A** | G | G/G |
| 3 | c.389 | **C/C** | T | T/T |
| 3 | c.435 | **C/C** | T | T/T |
| 3 | c.439 | **C/C** | A | A/A |
| 3 | c.472 | **C/C** | G | G/G |
| 3 | c.485 | **A/A** | G | G/G |
| 3 | c.497 | **T/T** | C | C/C |
| 3 | c.512 | **G/G** | C | C/C |
| 4 | c.517 | **A/A** | C | A/A |
| 4 | c.522 | **C/C** | G | C/C |
| 4 | c.532 | **G/G** | A | G/G |
| 4 | c.582 | **C/T** | C | C/C |
| 4 | c.594 | **T/T** | C | T/T |
| 4 | c.597 | **C/C** | G | C/C |
| 4 | c.601-603 |  | AAC | AAC/del |
| 4 | c.610 | **C/C** | T | T/C |
| 4 | c.611 | **A/A** | G | G/A |
| 4 | c.618 | **T/T** | C | C/T |
| 4 | c.619 | **G/G** | A | A/G |
| 4 | c.635 | **C/C** | T | T/T |
| 4 | c.636 | **A/A** | G | G/G |
| 4 | c.641 | **A/A** | G | G/G |
| 4 | c.643 | **A/A** | T | T/T |
| 4 | c.645 | **A/A** | T | T/T |
| 4 | c.655 | **C/C** | G | G/G |
| 4 | c.656 | **A/A** | C | C/C |
| 4 | c.667 | **C/C** | G | G/G |
| 5 | c.683 | **A/A** | G | G/G |
| 5 | c.684 | **C/C** | T | T/T |
| 5 | c.694 | **A/A** | G | G/G |
| 5 | c.782 | **G/G** | A | A/A |
| 5 | c.813 | **T/T** | C | C/C |
| 5 | c.828 | **T/T** | C | C/C |
| 5 | c.836 | **T/T** | A | A/A |
| 6 | c.867 | **G/T** | A | A/A |
| 6 | c.919 | **G/G** | T | T/T |
| 6 | c.920 | **C/C** | T | T/T |
| 6 | c.921 | **C/C** | T | T/T |
| 6 | c.924 | **T/T** | C | C/C |
| 6 | c.930 | **G/G** | G | G/A |
| 6 | c.942 | **A/A** | G | G/G |
| 6 | c.945 | **A/A** | C | C/C |
| 6 | c.946 | **G/G** | A | A/A |
| 7 | c.1012 | **A/A** | G | G/G |
| 7 | c.1064 | **G/G** | A | A/A |
| 7 | c.1109 | **G/G** | C | C/C |
| 7 | c.1117 | **G/G** | A | A/A |
| 7 | c.1123 | **G/G** | C | C/C |
| 7 | c.1128 | **C/C** | T | T/T |
| 7 | c.1130 | **C/C** | T | T/T |
| 7 | c.1131 | **G/G** | A | A/A |
| 7 | c.1143 | **G/G** | A | A/A |
| 7 | c.1176 | **T/T** | C | C/T |
| 7 | c.1181 | **A/A** | G | G/G |
| 8 | c.1185 | **C/G** | G | G/G |
| 8 | c.1198 | **C/A** | A | A/A |
| 8 | c.1290 | **C/C** | C | T/T |
| 9 | c.1457 | **C/C** | C | C/T |
| 9 | c.1459 | **C/C** | C | C/G |
| 9 | c.1470 | **G/G** | G | G/A |
| 9 | c.1471 | **G/G** | G | G/T |
| 9 | c.1485 | **C/C** | C | C/T |
| 9 | c.1486 | **T/T** | T | T/A |

**Supplemental Table 7: List of all the polymorphisms found in the CYP2D50-1 gene sequenced from 72 horses (144 alleles)**. Mutations with a frequency equal or higher than 50% of the total horses are highlighted in bold. The polymorphism highlighted in grey refers to the CYP2D50-1 converted structure for exons 8 and 9 where the T>C mutation is carried over from CYP2D50-2.

| Nucleotide substitution | Genomic position from ATG | Transcript position | Aminoacidic change | total alleles containing the SNP | total horses containing the SNP | Frequency alleles containing the SNP (%) | Frequency horses containing the SNP (%) | rs number |
| --- | --- | --- | --- | --- | --- | --- | --- | --- |
| G>A | g.92 | c.92 | R31H | 5 | 5 | 3.47 | 6.94 |  |
| A>C | g.102 | c.102 | P34P | 2 | 2 | 1.39 | 2.78 |  |
| C>A | g.111 | c.111 | P37P | 31 | 30 | 21.53 | 41.67 | rs1147575545 |
| G>A | g.134 | c.134 | G45E | 1 | 1 | 0.69 | 1.39 |  |
| T>G | g.235 | / | / | 31 | 30 | 21.53 | 41.67 | rs1149915697 |
| T>C | g.314 | / | / | 2 | 2 | 1.39 | 2.78 | rs1148598554 |
| G>C | **g.333** | **/** | **/** | **98** | **63** | **68.06** | **87.50** | **rs395827267** |
| G>A | g.398 | / | / | 1 | 1 | 0.69 | 1.39 | rs1152325391 |
| G>C | g.402 | / | / | 1 | 1 | 0.69 | 1.39 | rs1140233872 |
| C>A | g.403 | / | / | 8 | 8 | 5.56 | 11.11 | rs1138230048 |
| G>T | g.501 | / | / | 1 | 1 | 0.69 | 1.39 |  |
| A>G | g.530 | / | / | 31 | 30 | 21.53 | 41.67 | rs1149636304 |
| G>A | g.597 | / | / | 1 | 1 | 0.69 | 1.39 | rs1140942227 |
| G>C | g.656 | / | / | 2 | 1 | 1.39 | 1.39 |  |
| C>A | g.657 | / | / | 1 | 1 | 0.69 | 1.39 |  |
| G>A | **g.694** | **/** | **/** | **115** | **65** | **79.86** | **90.28** | **rs394908832** |
| C>T | **g.819** | **/** | **/** | **50** | **39** | **34.72** | **54.17** | **rs396623933** |
| A>G | **g.1088** | **/** | **/** | **108** | **63** | **75.00** | **87.50** | **rs1147786011** |
| T>C | **g.1096** | **/** | **/** | **144** | **72** | **100.00** | **100.00** |  |
| T>A | g.1130 | / | / | 34 | 21 | 23.61 | 29.17 | rs1146863177 |
| G>A | **g.1556** | **/** | **/** | **42** | **36** | **29.17** | **50.00** | **rs1141730935** |
| G>T | g.1595 | / | / | 2 | 2 | 1.39 | 2.78 | rs1151078947 |
| G>A | g.1697 | / | / | 8 | 8 | 5.56 | 11.11 | rs1145501538 |
| A>C | g.1966 | c.517 | R173R | 8 | 8 | 5.56 | 11.11 | rs1149865996 |
| C>G | g.1971 | c.537 | A179A | 8 | 8 | 5.56 | 11.11 | rs1148754385 |
| C>T | **g.2031** | **c.582** | **F194F** | **53** | **44** | **36.81** | **61.11** | **rs394409862** |
| C>T | g.2201 | / | / | 1 | 1 | 0.69 | 1.39 |  |
| G>A | g.2487 | c.687 | A229A | 1 | 1 | 0.69 | 1.39 |  |
| G>A | g.2511 | c.711 | P237P | 2 | 2 | 1.39 | 2.78 | rs1148105306 |
| C>A | g.2605 | c.805 | Q269K | 2 | 2 | 1.39 | 2.78 | rs1148503187 |
| T>C | **g.2703** | **/** | **/** | **97** | **62** | **67.36** | **86.11** | **rs69462655** |
| C>T | g.2775 | / | / | 1 | 1 | 0.69 | 1.39 | rs1140897437 |
| G>A | g.2803 | / | / | 37 | 31 | 25.69 | 43.06 | rs1136575479 |
| G>T | **g.2841** | **c.867** | **P289P** | **51** | **43** | **35.42** | **59.72** | **rs395461001** |
| T>C | g.3075 | / | / | 2 | 2 | 1.39 | 2.78 | rs1142628897 |
| G>T | g.3076 | / | / | 2 | 2 | 1.39 | 2.78 | rs1140129723 |
| G>A | g.3170 | / | / | 2 | 2 | 1.39 | 2.78 |  |
| C>T | g.3284 | c.1083 | A361A | 1 | 1 | 0.69 | 1.39 |  |
| G>C | g.3328 | c.1127 | G376A | 1 | 1 | 0.69 | 1.39 |  |
| G>T | g.3362 | c.1161 | V387V | 2 | 2 | 1.39 | 2.78 | rs1146471037 |
| G>A | g.3405 | / | / | 2 | 2 | 1.39 | 2.78 | rs1141488288 |
| G>T | **g.3521** | **/** | **/** | **51** | **42** | **35.42** | **58.33** | **rs395396474** |
| G>C | g.3702 | / | / | 3 | 2 | 2.08 | 2.78 | rs1147818029 |
| T>C | **g.3805** | **/** | **/** | **42** | **36** | **29.17** | **50.00** |  |
| C>T | g.3845 | / | / | 9 | 8 | 6.25 | 11.11 | rs1136481270 |
| C>T | g.3962 | c.1290 | F430F | 2 | 2 | 1.39 | 2.78 | rs1148456045 |
| C>A | g.4192 | c.1423 | Q475K | 2 | 2 | 1.39 | 2.78 | rs1137288236 |
| C>T | g.4226 | c.1457 | T486I | 11 | 11 | 7.64 | 15.28 |  |
| C>G | g.4228 | c.1459 | L487V | 11 | 11 | 7.64 | 15.28 |  |
| C>T | g.4235 | c.1466 | S489F | 1 | 1 | 0.69 | 1.39 |  |
| G>A | g.4239 | c.1470 | P490P | 11 | 11 | 7.64 | 15.28 | rs394549514 |
| T>G | **g.4240** | **c.1471** | **S491A** | **61** | **42** | **42.36** | **58.33** | **rs396861920** |
| C>T | g.4254 | c.1485 | L495L | 11 | 11 | 7.64 | 15.28 | rs396183283 |
| T>A | g.4255 | c.1486 | C496S | 11 | 11 | 7.64 | 15.28 | rs394932323 |
| Exons 8 & 9 conversion | | | | 42 | 36 | 29.17 | 50.00 |  |

**Supplemental Table 8: List of all the polymorphisms found in the CYP2D82 gene sequenced from 72 horses (144 alleles).** Mutations with a frequency equal or higher than 50% of the total horses are highlighted in bold. The two mutations highlighted in grey are not confirmed to be on the same allele and therefore the aminoacidic change is only hypothetic and cannot be confirmed (the two mutations were only seen in 2 horses in heterozygosis).

| Nucleotide substitution | Genomic position from ATG (NC_009171.3) | Transcript position | Aminoacidic change | Alleles containing the SNP | Horses containing the SNP | Frequency alleles containing the SNP (%) | Frequency horses containing the SNP (%) | rs number |
| --- | --- | --- | --- | --- | --- | --- | --- | --- |
| A>G | g.6 | c.6 | G2G | 1 | 1 | 0.69 | 1.39 | rs1144458724 |
| G>A | g.33 | c.33 | P11P | 14 | 14 | 9.72 | 19.44 | rs1146825900 |
| G>A | g.83 | c.83 | R28Q | 26 | 20 | 18.06 | 27.78 | rs1141439398 |
| C>T | g.85 | c.85 | R29C | 1 | 1 | 0.69 | 1.39 | rs1150936359 |
| G>A | g.201 | / | / | 1 | 1 | 0.69 | 1.39 | rs1147439497 |
| C>T | g.247 | / | / | 8 | 8 | 5.56 | 11.11 | rs1146986318 |
| C>T | **g.271** | **/** | **/** | **49** | **36** | **34.03** | **50.00** | **rs69449227** |
| C>A | g.289 | / | / | 25 | 19 | 17.36 | 26.39 | rs1150705697 |
| G>T | g.301 | / | / | 1 | 1 | 0.69 | 1.39 |  |
| C>T | g.302 | / | / | 1 | 1 | 0.69 | 1.39 | [rs1141814869](https://www.ensembl.org/Equus_caballus/Variation/Explore?db=core;g=ENSECAG00000031996;r=28:39836761-39836842;source=dbSNP;v=rs1141814869;vdb=variation;vf=5053981) |
| A>G | g.303 | / | / | 1 | 1 | 0.69 | 1.39 | [rs1137234147](https://www.ensembl.org/Equus_caballus/Variation/Explore?db=core;g=ENSECAG00000031996;r=28:39836761-39836842;source=dbSNP;v=rs1137234147;vdb=variation;vf=5069687) |
| G>T | g.333 | / | / | 1 | 1 | 0.69 | 1.39 | rs1140606639 |
| TG del | g.841-842 | / | / | 1 | 1 | 0.69 | 1.39 |  |
| G>A | g.1005 | c.295 | E99E | 1 | 1 | 0.69 | 1.39 |  |
| G>A | **g.1119** | **/** | **/** | **49** | **36** | **34.03** | **50.00** | **rs69449226** |
| G>A | g.1241 | / | / | 8 | 6 | 5.56 | 8.33 |  |
| C>G | g.1242 | / | / | 7 | 5 | 4.86 | 6.94 |  |
| A>G | g.1245 | / | / | 8 | 6 | 5.56 | 8.33 |  |
| G>C | g.1250 | / | / | 8 | 6 | 5.56 | 8.33 |  |
| A>C | g.1251 | / | / | 8 | 6 | 5.56 | 8.33 |  |
| C>T | g.1252 | / | / | 8 | 6 | 5.56 | 8.33 |  |
| C>A | g.1256 | / | / | 8 | 6 | 5.56 | 8.33 |  |
| C>A | g.1269 | / | / | 22 | 18 | 15.28 | 25.00 | rs1148727171 |
| C>T | g.1277 | / | / | 8 | 6 | 5.56 | 8.33 |  |
| C>G | g.1279 | / | / | 8 | 6 | 5.56 | 8.33 |  |
| G>A | g.1291 | / | / | 1 | 1 | 0.69 | 1.39 |  |
| G>A | **g.1307** | **/** | **/** | **48** | **35** | **33.33** | **48.61** | **rs393740984** |
| A>G | g.1320 | / | / | 9 | 7 | 6.25 | 9.72 | rs1151331102 |
| A>C | g.1347 | / | / | 8 | 6 | 5.56 | 8.33 | rs1145899946 |
| G>C | g.1418 | / | / | 7 | 7 | 4.86 | 9.72 |  |
| T>C | g.1498 | / | / | 6 | 3 | 4.17 | 4.17 | rs394880199 |
| A>G | g.1505 | / | / | 17 | 14 | 11.81 | 19.44 | rs397495312 |
| A>G | g.1518 | / | / | 6 | 5 | 4.17 | 6.94 |  |
| T>A | g.1563 | / | / | 7 | 5 | 4.86 | 6.94 | rs1137932152 |
| C>A | g.1607 | / | / | 2 | 2 | 1.39 | 2.78 |  |
| G>C | **g.1875** | **c.363** | **G121G** | **49** | **36** | **34.03** | **50.00** |  |
| C>G | g.1888 | c.376 | R126G | 18 | 15 | 12.50 | 20.83 |  |
| A>C | g.1892 | c.380 | Y127S | 2 | 2 | 1.39 | 2.78 |  |
| T>C | **g.1951** | **c.439** | **L147L** | **100** | **60** | **69.44** | **83.33** | **rs1139878064** |
| G>C | g.1984 | c.472 | E158Q | 8 | 8 | 5.56 | 11.11 | rs1143153764 |
| A>C | **g.2046** | **/** | **/** | **99** | **60** | **68.75** | **83.33** |  |
| A>C | **g.2055** | **/** | **/** | **79** | **54** | **54.86** | **75.00** |  |
| G>A | **g.2056** | **/** | **/** | **79** | **54** | **54.86** | **75.00** |  |
| G>A | **g.2059** | **/** | **/** | **99** | **60** | **68.75** | **83.33** |  |
| C>T | g.2105 | / | / | 7 | 5 | 4.86 | 6.94 | rs1151570010 |
| T>G | g.2214 | c.611 | L204R | 8 | 7 | 5.56 | 9.72 | rs1138659802 |
| T>C | g.2226 | c.623 | L208L | 2 | 2 | 1.39 | 2.78 |  |
| C>T | g.2228 | c.625 | L209L | 8 | 7 | 5.56 | 9.72 | rs1143077913 |
| C>T | g.2245 | c.642 | D214D | 1 | 1 | 0.69 | 1.39 |  |
| AC ins | g.2294 | / | / | 14 | 14 | 9.72 | 19.44 |  |
| T>C | **g.2399** | **/** | **/** | **49** | **36** | **34.03** | **50.00** | **rs1149090683** |
| A>T | **g.2406** | **/** | **/** | **49** | **36** | **34.03** | **50.00** | **rs1136278894** |
| T>C | g.2446 | / | / | 1 | 1 | 0.69 | 1.39 | rs1137401693 |
| G>A | g.2519 | / | / | 21 | 44 | 14.58 | 61.11 | rs395351837 |
| C>T | g.2628 | c.697 | L233F | 2 | 2 | 1.39 | 2.78 |  |
| A>G | g.2635 | c.704 | H235R | 2 | 2 | 1.39 | 2.78 | rs1147081710 |
| G>C | g.2646 | c.715 | V239L | 1 | 1 | 0.69 | 1.39 |  |
| C>T | g.2650 | c.719 | A240V | 1 | 1 | 0.69 | 1.39 |  |
| T>C | g.2651 | c.720 | A240A | 1 | 1 | 0.69 | 1.39 |  |
| A>G | g.2673 | c.742 | K248E | 8 | 6 | 5.56 | 8.33 | rs1145722969 |
| A>G | **g.2674** | **c.743** | **K248R** | **79** | **51** | **54.86** | **70.83** | **rs1143545655** |
| G>A | g.2675 | c.744 | K248K | 8 | 6 | 5.56 | 8.33 | rs1148458062 |
| AAG>GGA | g.2673-2675 | c.742-744 | K248G | 4 | 2 | 2.78 | 2.78 |  |
| C>G | **g.2691** | **c.760** | **L254V** | **98** | **59** | **68.06** | **81.94** | **rs394128659** |
| C>G | **g.2703** | **c.772** | **L258V** | **49** | **36** | **34.03** | **50.00** |  |
| A>G | g.2733 | c.802 | T268A | 2 | 2 | 1.39 | 2.78 | rs1136055998 |
| C>G | **g.2735** | **c.804** | **T268T** | **83** | **55** | **57.64** | **76.39** | **rs1151886597** |
| ACC>GCG | g.2733-2735 | c.802-804 | T268A | 2 | 2 | 1.39 | 2.78 |  |
| C>T | **g.2744** | **c.813** | **P271P** | **47** | **35** | **32.64** | **48.61** | **rs1139435570** |
| T>C | **g.2765** | **c.834** | **F278F** | **99** | **60** | **68.75** | **83.33** | **rs395257132** |
| T>A | g.2767 | c.836 | L279Q | 2 | 2 | 1.39 | 2.78 | rs1142815816 |
| C>T | g.3013 | c.897 | R299R | 18 | 15 | 12.50 | 20.83 |  |
| C>G | g.3064 | c.948 | T316T | 15 | 12 | 10.42 | 16.67 |  |
| TCC del | g.3245-3247 | / | / | 32 | 26 | 22.22 | 36.11 |  |
| C>G | g.3331 | / | / | 2 | 2 | 1.39 | 2.78 |  |
| T>C | **g.3403** | **c.1063** | **C355R** | **98** | **60** | **68.06** | **83.33** | **rs396631299** |
| T>G | g.3469 | c.1129 | L377V | 14 | 14 | 9.72 | 19.44 | rs1148651933 |
| A>G | g.3472 | c.1132 | T378A | 14 | 14 | 9.72 | 19.44 | rs1136958956 |
| CCACATGACAT del | g.3474-3484 | c.1134-1144 | 378 fs | 1 | 1 | 0.69 | 1.39 |  |
| A>G | **g.3493** | **c.1153** | **I385V** | **99** | **60** | **68.75** | **83.33** | **rs395645145** |
| C>G | g.3539 | / | / | 2 | 2 | 1.39 | 2.78 | rs1141582269 |
| A>G | **g.3644** | **/** | **/** | **51** | **37** | **35.42** | **51.39** |  |
| G>C | **g.3776** | **/** | **/** | **51** | **37** | **35.42** | **51.39** |  |
| G>C | g.3889 | / | / | 7 | 5 | 4.86 | 6.94 | rs1144530662 |
| A>C | g.3919 | / | / | 32 | 26 | 22.22 | 36.11 | rs1139711456 |
| C>G | **g.4010** | **/** | **/** | **105** | **62** | **72.92** | **86.11** | **rs394904453** |
| T>C | g.4018 | / | / | 1 | 1 | 0.69 | 1.39 |  |
| C>T | g.4104 | c.1249 | R417C | 32 | 26 | 22.22 | 36.11 | rs1148309512 |
| G>A | g.4141 | c.1286 | R429H | 8 | 8 | 5.56 | 11.11 | rs1145664752 |
| G>A | g.4253 | / | / | 1 | 1 | 0.69 | 1.39 |  |
